# Identification of lipid biomarker from serum in patients with chronic obstructive pulmonary disease

**DOI:** 10.1186/s12931-020-01507-9

**Published:** 2020-09-21

**Authors:** Ding Liu, Maureen Meister, Shiying Zhang, Chi-In Vong, Shuaishuai Wang, Ruixie Fang, Lei Li, Peng George Wang, Pierre Massion, Xiangming Ji

**Affiliations:** 1 Department of Chemistry, Georgia State University, Atlanta, GA 30303 USA; 2 Department of Nutrition, Georgia State University, Atlanta, 30302 USA; 3 Department of Mathematics and Statistics, Georgia State University, Atlanta, GA 30302 USA; 4 Cancer Early Detection and Prevention Initiative, Vanderbilt Ingram Cancer Center; Division of Allergy, Pulmonary and Critical Care Medicine, Vanderbilt University School of Medicine, Nashville, TN 37232 USA

**Keywords:** Chronic obstructive pulmonary disease (COPD), Lipidomics, Biomarkers, OPLS-DA, Receiver operating characteristic

## Abstract

**Background:**

Chronic obstructive pulmonary disease (COPD) is the third leading cause of death in the United States with no effective treatment. The current diagnostic method, spirometry, does not accurately reflect the severity of COPD disease status. Therefore, there is a pressing unmet medical need to develop noninvasive methods and reliable biomarkers to detect early stages of COPD. Lipids are the fundamental components of cell membranes, and dysregulation of lipids was proven to be associated with COPD. Lipidomics is a comprehensive approach to all the pathways and networks of cellular lipids in biological systems. It is widely used for disease diagnosis, biomarker identification, and pathology disorders detection relating to lipid metabolism.

**Methods:**

In the current study, a total of 25 serum samples were collected from 5 normal control subjects and 20 patients with different stages of COPD according to the global initiative for chronic obstructive lung disease (GOLD) (GOLD stages I ~ IV, 5 patients per group). After metabolite extraction, lipidomic analysis was performed using electrospray ionization mass spectrometry (ESI-MS) to detect the serum lipid species. Later, the comparisons of individual lipids were performed between controls and patients with COPD. Orthogonal projections to latent structures discriminant analysis (OPLS-DA) and receiver operating characteristic (ROC) analysis were utilized to test the potential biomarkers. Finally, correlations between the validated lipidomic biomarkers and disease stages, age, FEV1% pack years and BMI were evaluated.

**Results:**

Our results indicate that a panel of 50 lipid metabolites including phospholipids, sphingolipids, glycerolipids, and cholesterol esters can be used to differentiate the presence of COPD. Among them, 10 individual lipid species showed significance (*p* < 0.05) with a two-fold change. In addition, lipid ratios between every two lipid species were also evaluated as potential biomarkers. Further multivariate data analysis and receiver operating characteristic (ROC: 0.83 ~ 0.99) analysis suggest that four lipid species (AUC:0.86 ~ 0.95) and ten lipid ratios could be potential biomarkers for COPD (AUC:0.94 ~ 1) with higher sensitivity and specificity. Further correlation analyses indicate these potential biomarkers were not affected age, BMI, stages and FEV1%, but were associated with smoking pack years.

**Conclusion:**

Using lipidomics and statistical methods, we identified unique lipid signatures as potential biomarkers for diagnosis of COPD. Further validation studies of these potential biomarkers with large population may elucidate their roles in the development of COPD.

## Introduction

Chronic obstructive pulmonary disease (COPD) is a chronic lung disease, which is characterized by irreversible airflow obstruction, mucus hypersecretion, and chronic airway inflammation. It is the third leading cause of death worldwide, and it was ranked second as disability-adjusted life-years (DALY) from 1990 to 2010 [1–3]. Previous studies demonstrated that the progressive structural changes in the bronchial epithelium are associated with sub-epithelial fibrous remodeling [4]. Tobacco smoking is the leading cause of COPD although other factors such as air pollution, genetic susceptibility and occupational exposure also account for COPD [5]. Spirometry is the major clinical test used to diagnose patients with COPD by measuring the airflow volumes into and out of the lung. Spirometry is clinically utilized to measure the forced expiratory volume in one second (FEV_1_) and forced vital capacity (FVC), the ratio of FEV_1_/FVC as the indicator of obstructive or restrictive ventilatory defects [6]. However, clinical evidence indicates that spirometry can neither predict the disease activity or the severity nor differentiate the subtypes of disease. Moreover, symptoms such as bronchial hyper-responsiveness and frequent exacerbations are also used as indicators of COPD disease progression. However, these clinical markers have no specificity and cannot be quantified [7]. Until now, there is no effective treatment for COPD. Therefore, novel biomarkers are urgently needed for early detection of COPD, which holds the potential for successful intervention in the progression of the disease [8].

Cellular lipid species play an essential role in various cellular processes, including membrane integrity, energy sources, and cellular signaling processes such as cell proliferation, metabolism, and apoptosis induction. Accumulating studies indicate that dysregulation of lipids is associated with the pathogenesis of COPD [9, 10]. Obesity with high compositions of triglyceridesis and cholesterol is associated with declining lung function and increased morbidity of COPD [11]. Meta-analysis shows that the use of statins was associated with 38% reduction in all-cause mortality (95% CI 0.52 to 0.73) and a 52% reduction in COPD mortality [12]. In addition, phospholipid is a significant component of cell membranes, which consists of a hydrophilic head and two hydrophobic fatty acid tails. Phospholipids account for 85% of pulmonary surfactant mass, and the composition and concentration of individual phospholipid are critical factors of functional pulmonary surfactants [13]. The more enhancement of phosphatidylcholine (PC)16:0/16:0 occurs within the surfactants, the surfactants are more resistant to the high pressures generated at the air-liquid interface of the mammalian lung [13]. In addition, total surfactant phospholipids are also known to decrease in patients with COPD, which may correlate with pulmonary function [14]. Moreover, dysregulation of bioactive lipids such as ceramides, sphingomyelins, and sphingosine-1-phospate (S1P) were associated with smoking. The amount of sphingolipids was significantly higher in smokers with COPD than in smokers without COPD [15, 16]. The S1P promotes acquired immune responses in smoke-induced lung inflammation and lymphocyte trafficking [17, 18]. Data from COPDGene cohort showed that three unique sphingolipids found in the plasma are strongly associated with emphysema and COPD exacerbation phenotypes, but not with airflow obstruction and chronic bronchitis [19]. Other lipids such as ceramide plays important roles in the induction of apoptosis and cellular senescence, and free fatty acids could cause similar inflammatory responses [20, 21]. These data suggest that lipids play important roles in the development of COPD, and a detailed lipidomic profiling across different stage of COPD for individual lipids may shed light on the pathogenesis of COPD. Lipidomics is an emerging “omics” approach, which comprehensively measures the full lipid components of cells, tissues, and organisms [22]. To determine whether lipid metabolites could be biomarkers for the diagnosis of COPD, global untargeted lipidomic analysis was performed to investigate serum samples from patients with different stages of COPD. Subsequently, general statistical analysis, orthogonal partial least squares discriminant analysis (OPLS-DA), and receiver operating characteristic (ROC) analysis were performed to assess the quantitative change of lipid metabolites. Finally, the correlation of lipid productions with ages and smoking status in patients was also assessed.

## Materials and methods

### Patient and control serum sample collection

In this study, 25 serum samples were obtained from the Vanderbilt Medical Center and the Nashville Veteran Affairs Medical Center. All procedures performed in studies involving human participants were in accordance with the ethical standards of the institutional and/or national research committee and with the 1964 Helsinki Declaration and its later amendments or comparable ethical standards. The study was approved by the local institutional review board (Vanderbilt University Medical Center and Veterans Affairs, Tennessee Valley Healthcare System, Nashville, Tennessee Institutional Review Boards), and informed consents were obtained from all individuals at both institutions. Individuals providing serum samples were grouped based on their FEV_1_ value, each group consisting of 5 individuals. Clinical information, sample size, classification of patients with COPD based on Global Initiative for Chronic Obstructive Lung Disease Stage [23], and normal control are listed in Table 1. From each subject, 1 mL of whole blood was collected into a vacutainer tube without anticoagulant. Each serum sample was promptly separated (no more than 4 h after collection of whole blood) and stored at − 80 °C immediately.

**Table 1:** Characteristics of Patients by Lipidomic Analysis

GOLD Status	No	I	II	III	IV
N=	5	5	5	5	5
**FEV1%**					
Average + Std	109 ± 3.67	77 ± 1.87	53.4 ± 3.21	47.4 ± 1.14	27.4 ± 3.64
**Age**					
Average + Std	65.81 ± 5.89	67 ± 4.30	72.6 ± 2.97	71.8 ± 8.53	65.8 ± 5.89
**Smoking**					
Yes	5/5 (100%)	5/5 (100%)	5/5 (100%)	5/5 (100%)	5/5 (100%)
**Pack-Years**					
Average + Std	49.4 ± 13.04	63 ± 19.26	65.9 ± 30.40	67.2 ± 29.31	69 ± 21.4
**BMI**					
Average + Std	26.4 ± 4.88	28.3 ± 4.30	26.3 ± 2.97	25.7 ± 8.53	24.6 ± 8.53
**Gender**					
Male	2/5 (40%)	2/5 (40%)	3/5 (60%)	4/5 (80%)	4/5 (80%)

### Lipids extraction from serum samples

Serum metabolite extraction was performed as described previously [24]. Briefly, 50 μL of each serum sample was mixed with 160 μL of chloroform and 320 μL of methanol containing BHT (50 μg/ml). After vortex mixing for 20 min, 160 μl of water was added and shook. Later, the sample was centrifuged for 5 min at 2000 g, after that the lower layer was retained. Then another 160 μL chloroform was added, shook and centrifuged, and the lower layer was collected and combined with the previous step. The combined lower layers were washed with a small volume of 1 M KCl solution once. For each sample, chloroform was used to dissolve the extracted lipids from serum (chloroform vs serum, 10:1 in volume), then 6 μL of the serum extraction lipid in chloroform was prepared for Waters Xevo instrument infusion in next step. All extracted samples were transported on dry ice to the Kansas Lipidomics Research Center (KLRC) for lipid analysis. Most of the phospholipid standards were purchased from Avanti Polar Lipids, Inc. (Alabaster, AL), except for di24:1-PE and di24:1-PG, which were prepared by transphosphatidylation of di24:1-PC [25].

### ESI-MS lipid profiling

In order to profile the lipid species, an automated electrospray ionization tandem mass spectrometry method was applied. In this method, the ratio of head groups plus total acyl carbons to total double bonds is used to determine the serum lipid species. Data acquisition and analysis were performed as described previously with modifications [26]. In short, proper amounts of internal standards, obtained and quantified as previously described [27], were added in the following quantities: 0.30 nmol lysophosphatidylglycerol—LPG(14:0), 0.30 nmol LPG (18:0), 0.30 nmol phosphatidylglycerol— PG (14:0/14:0), 0.30 nmol lysophosphatidylethanolamine—LPE(14:0), 0.30 nmol LPE(18:0), 0.30 nmol phosphatidylethanolamine—PE(12:0/12:0), 0.30 nmol PE(23:0/23:0), 0.60 nmol lysophosphatidylcholine—LPC(13:0), 0.60 nmol LPC(19:0), 0.60 nmol phosphatidylcholine—PC(12:0/12:0), 0.60 nmol PC(24:1/24:1), 0.30 nmol lysophosphatidic acid—LPA(14:0), 0.30 nmol LPA(18:0), 0.30 nmol lysophosphatidic acidcPA(20:0/20:0), 0.30 nmol PA(14:0/14:0), 0.20 nmol phosphatidylserine—PS(14:0/14:0), 0.29 nmol phosphatidylinositol—PI (16:0/18:0), 0.11 nmol PI (18:0/18:0), 10 nmol cholesterol ester—CE(13:0) and 10 nmol CE(23:0). The 6 μL of the serum extraction lipid from the last step was mixed with the standard internal mixture and finally dissolved in 1.2 ml solvent, which was 300 mM ammonium acetate/chloroform/methanol with the ratio 300/665/35. The mixture was introduced by continuous infusion into the ESI source on a triple quadrupole MS/MS (API 4000, Applied Biosystems, Foster City, CA), using an auto-sampler (LC Mini PAL, CTC Analytics AG, Zwingen, Switzerland) fitted with a 300 μL loop for the acquisition time and pumped to the ESI needle at 30 ml/min. The collision gas pressure was set at 2 (arbitrary units). The collision energies, with nitrogen in the collision cell, were + 5 V for CE, + 28 V for PC, + 5 V for PA, + 12 V for PE, + 17 V for PI and + 13 V for PS. The cone voltage was + 24 V for CE and + 40 V for all other species. The source temperature (heated nebulizer) was 150 °C, and the interface heater was on, cone gas flow was 150 L per hour, desolation gas flow was 650 L per hour, the gas flow was 0.14 ml per min, scan rate was set to 200 units per sec.

All scans were performed in a positive mode. Lipid species with a common head group fragment were determined by the precursor ion and neutral loss scans of the lipid extracts. Lipid species were detected with the following scans: CE, [M + NH4]^+^ with precursor of 369.3 (Pre 369.3); PC and LPC, [M + H]^+^ with Pre 184.1; PA, [M + NH4]^+^ with Neutral Loss of 115.0 (NL 115.0); PE and LPE, [M + H]^+^ ions with NL 141.0; PI, [M + NH4]^+^ with NL 277.0; PS, [M + H]^+^ with NL 185.0; Triacylglycerol (TAG) with ten NL, including palmitic (P, 16:0), linolenic (Ln, 18:3), linoleic (L, 18:2), oleic (O, 18:1), stearic (S, 18:0), eicosadienoic (20:2), gadoleic (20:1), arachidic (20:0), erucic (22:1), and behenic (22:0). PG and LPG were determined by the same diacyl standards with other PC and PE species, but no response factors were reported.

Deconvolution is applied to the data for overlapping of isotopic peaks. Each spectrum was subtracted by background. Later, the signal normalized by internal standards was used for relative quantification. Since molecular species in the same class would have similar ionization efficiency, the same class of internal standards was applied to quantification analysis. Quality controls were made by pooling from all 25 samples. After the first 6 quality controls, the mass spectra of internal standard mixture only, one quality control, and normally 10 patients’ samples were acquired successively. The average molar amount of lipid species detected in “internal standards only” was used for background subtract. At last, the data were normalized by the analyzed sample amount to generate data with the concentration unit of nmol/μL.

### Statistical analysis

Four criteria were applied to select lipid biomarkers candidates. Species below the detection of limit, species with excessive missing values, species with insignificant difference between patient and control groups, and species with insignificant fold change would be removed. Criteria for retention were: 1) mean of serum lipid with concentration was higher than 0.0007 nmol/μL; 2) lipid species with less than 50% missing value; 3) difference between control and patient groups was statistically significant *p* < 0.05; 4) fold changes in concentration in serum lipid is > 2 (up or down). Later, Orthogonal Projections to Latent Structures (OPLS) and receiver operating characteristic curve (ROC) were applied to evaluate the biomarker candidates. In MetaboanalystR, the OPLS-DA model was constructed, and ROC analysis was performed based on the selected biomarker candidates [28]. All the results were demonstrated as the mean ± SD. Individual *p*-values were reported in the figures with values of *p* < 0.05 considered as statistically significant.

## Results

### Subject characteristics

Previous data demonstrated that patients with COPD exhibit systematic dysregulation in lipid metabolism, in which sphingolipid pathway is higher in smokers with COPD compared with smokers without COPD [15]. Other study indicated that sphingomyelins are strongly associated with emphysema and glycosphingolipids are associated with COPD exacerbations [19]. To confirm these findings and search for novel lipid biomarkers associated with COPD, we examined the serum lipid profiling from 25 serum samples, among them 20 samples from patients with different severity levels of COPD and 5 samples as controls showed in Table 1. Each GOLD stage has 5 samples from the subjects. There were no significant differences in baseline characteristics between the patients with and without COPD. All the subjects have a history of smoking and were of similar age. Not surprisingly, there is a positive correlation between the pack-years (the number of cigarette smoking packs multiplied by the number of smoking years within same patients) and the stage of COPD.

### Lipid profiling of 377 lipid species

**Fig. 1:**
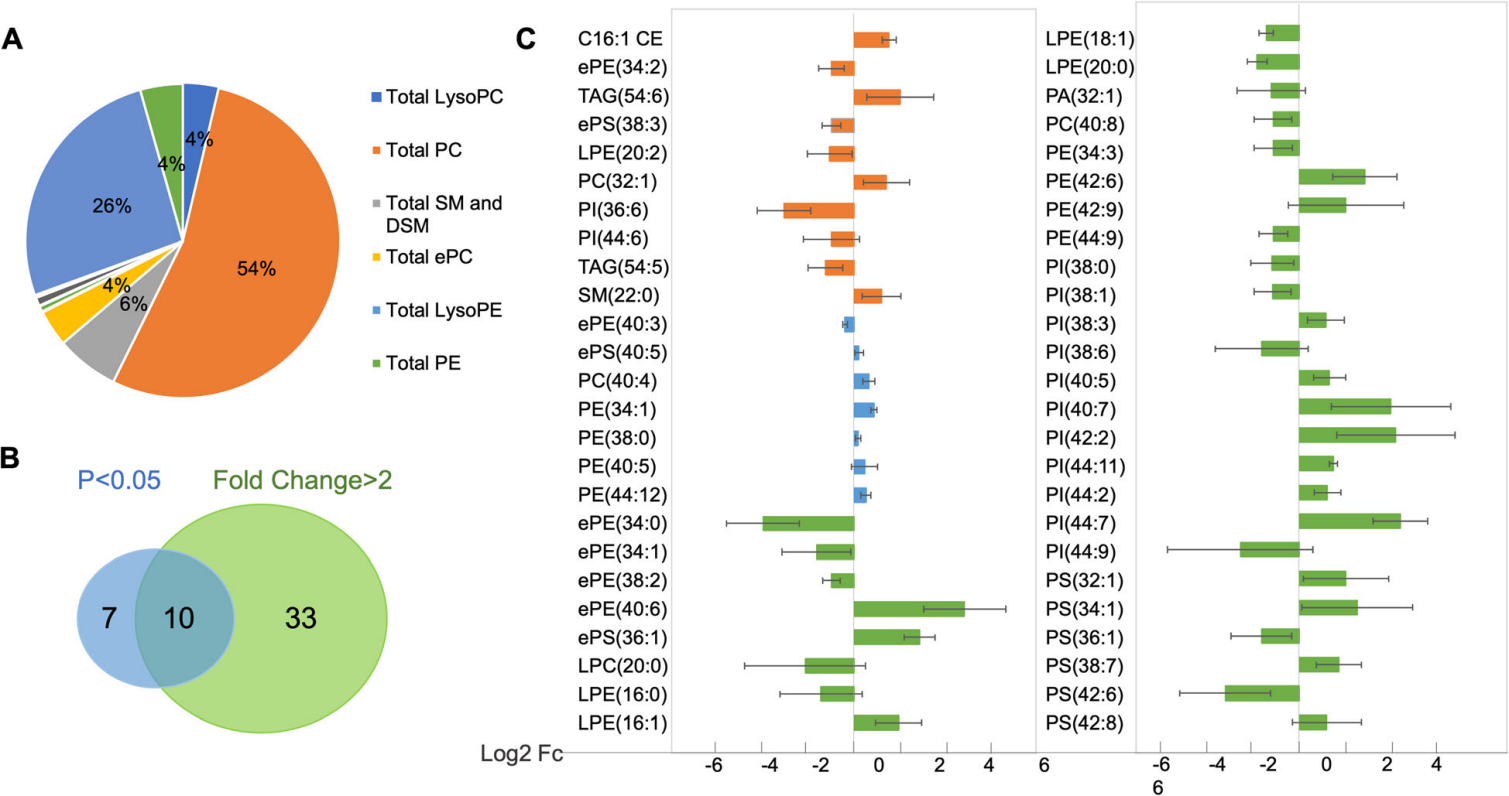
Lipidomic analysis of serum samples from patients with different severity of COPD. **a**. Relative lipid species composition in serum samples by electrospray ionization mass spectrometry (ESI-MS); **b**. Venn diagram of 50 lipid species with either FDR-adjusted significance (*p* < 0.05, blue with 17 lipid species) or two-times fold change (green with 43 lipids species); **c**. Detailed fold change of 50 lipid species: 10 lipid species with both two-times fold change and p < 0.05 (orange bar), 32 lipid species with two-times fold change only (green bar), 7 lipid species with significant change (blue bar) only

Using electrospray ionization mass spectrometry (ESI-MS), 377 individual lipid species were identified using previous published method (Supplemental Table 1) [29]. These lipid species include 11 classes of phospholipids, 1 class of sphingolipids, 1 class of sterol lipids, and 1 class of glycerolipids. Among the abundance of the lipid species, our lipidomics data indicates that 54% of PC, 26.22% of CE, 6.5% of SM, 4.4% of TAG, 10% of other lipid species (Fig. 1a). Among all identified lipids species, the highest concentration in serum was CE (18:2) (112.25 ± 33.50 mM in patients vs 118.34 ± 33.02 mM in controls). Among phospholipids, the lipid with the highest concentration in serum was PC (34:2) (91.68 ± 27.10 mM in patients, 103.92 ± 49.07 mM in controls). Among sphingolipids, the lipid species with the highest concentration in serum was SM (16:0) (17.64 ± 4.97 in patients, 17.69 ± 3.01 mM in controls). Among triacylglycerol species, the lipid with the highest concentration in serum was TAG (52:2) (13.36 ± 7.63 mM in patients, 14.40 ± 8.2 mM in controls). Comparing the concentration of all the lipid species in serum, the significant fold change between patient and control groups ranged from positive 40-fold for ePS (36:3) to negative 33.3-fold for ePE (40:6). Supplemental Figure 1 shows the examples of mass spectrum of precursor and neutral loss scan.

### Identification of individual lipid species as biomarker candidate

In order to quantify the individual lipid species, the concentrations of individual lipid species with more than 50% detection values were retained for further analysis (total 268 species). The remaining missing values were replaced by the half of the minimum positive value in original data. Log transformation was performed before statistics analysis. Fold changes and *p*-value were also applied to separate the individual lipid species among the controls and patients with different stages of COPD. First, a t-test was applied to compare the difference between the mean concentration of lipid species in COPD and controls. As shown in Fig. 1b, a total of 17 lipid species were identified to be statistically significant different (*p* < 0.05). Later, the Log2 value of fold change was used as the cut-off value to compare the lipid species from disease and from control groups (Log2Fc > 1 or < − 1). Of interest, 43 lipids were obtained to have distinct concentration values of lipid species (Fig. 1b). Therefore, based on the statistical approach, a total of 50 lipid species were selected as biomarker candidates. As shown in Fig. 1c, among these 50 lipid species, 33 lipid species had considerable fold change but *p* > 0.05 (green bar), 7 lipid species had statistically significant difference but fold change < 2 (blue bar), and 10 lipid species met both criteria as fold change > 2 and *p* < 0.05 (orange bar). These results demonstrate a total of 10 lipid species have satisfied both *p*-value and fold change cut-off and could be selected as potential biomarker candidates.

### Identification of lipid species ratio as biomarker candidate

Previous data suggest that the ratios of the concentrations between two relative metabolites could convey more information compared to the two individual metabolites [30]. In addition, the ratios between two or more lipid species especially between the subjects and products could be a biomarker for diseases which reflecting the specific metabolic enzyme [31]. For example, the ratio of lysophosphatidylcholine (LPC) to phosphatidylcholine (PC) was indicated the dysregulation of lysophosphatidylcholine acyltransferase 1, that play essential roles during the tumorigenic process of gastric cancer [32]. In this study, within the 268 lipid species after excluding missing values, ratios of all possible lipid pairs were calculated, and the top 10 ratios (based on *p*-value) were chosen as lipid ratio candidates for further biomarker analysis. In Table 2, the top 10 lipid ratio candidates with p-value < 0.001 and fold change > 2 were demonstrated. Interestingly, the most significant ones were the ratio of glycerophospholipids to cholesterol ester such as PI (38:4)/C16:1 CE and PI(36:2)/C16:1 CE. Moreover, 8 of the ratios of glycerophospholipids to cholesterol ester were lower in COPD groups, while the rest 2 lipid ratios biomarkers were higher in COPD groups. Patients with severe COPD have a higher level of cholesterol in the blood [33]. The upregulation of cholesterol could be partially due to the adverse effects of cigarette smoking on lipoprotein regulation [34–37]. In aggregate, we successfully identified 10 lipid ratio biomarker candidates from the patients with different severity of COPD.

**Table 2:** Top 10 lipid ratio biomarker candidates

Rank	Lipid Species	Trend	Lipid Class	AUC	Log2FC	*p* value
1	PI(38:4)/C16:1 CE	↓	glycerophospholipids/sterol lipids	1	−1.59	6.41E-05
2	PI(36:2)/C16:1 CE	↓	glycerophospholipids/sterol lipids	0.98	−2.01	2.25E-04
3	C16:1 CE/C19:0 CE	↑	sterol lipids/sterol lipids	0.98	1.24	3.72E-05
4	ePC(38:2)/C16:1 CE	↓	glycerophospholipids/sterol lipids	0.97	−1.8	2.26E-05
5	LPC(18:0)/C20:3 CE	↓	glycerophospholipids/sterol lipids	0.95	−1.13	3.38E-05
6	LPC(16:1)/C16:1 CE	↓	glycerophospholipids/sterol lipids	0.94	−1.43	1.87E-04
7	PC(32:0)/C16:1 CE	↓	glycerophospholipids/sterol lipids	0.94	−1.23	1.51E-04
8	PC(34:3)/C16:1 CE	↓	glycerophospholipids/sterol lipids	0.94	−1.36	1.00E-04
9	PC(38:1)/C16:1 CE	↓	glycerophospholipids/sterol lipids	0.94	−1.78	1.17E-04
10	PC(40:4)/ePC(38:2)	↑	glycerophospholipids/glycerophospholipids	0.94	1.14	8.76E-05

### Evaluation of lipid biomarker candidates by OPLS-DA and ROC analysis

**Fig. 2:**
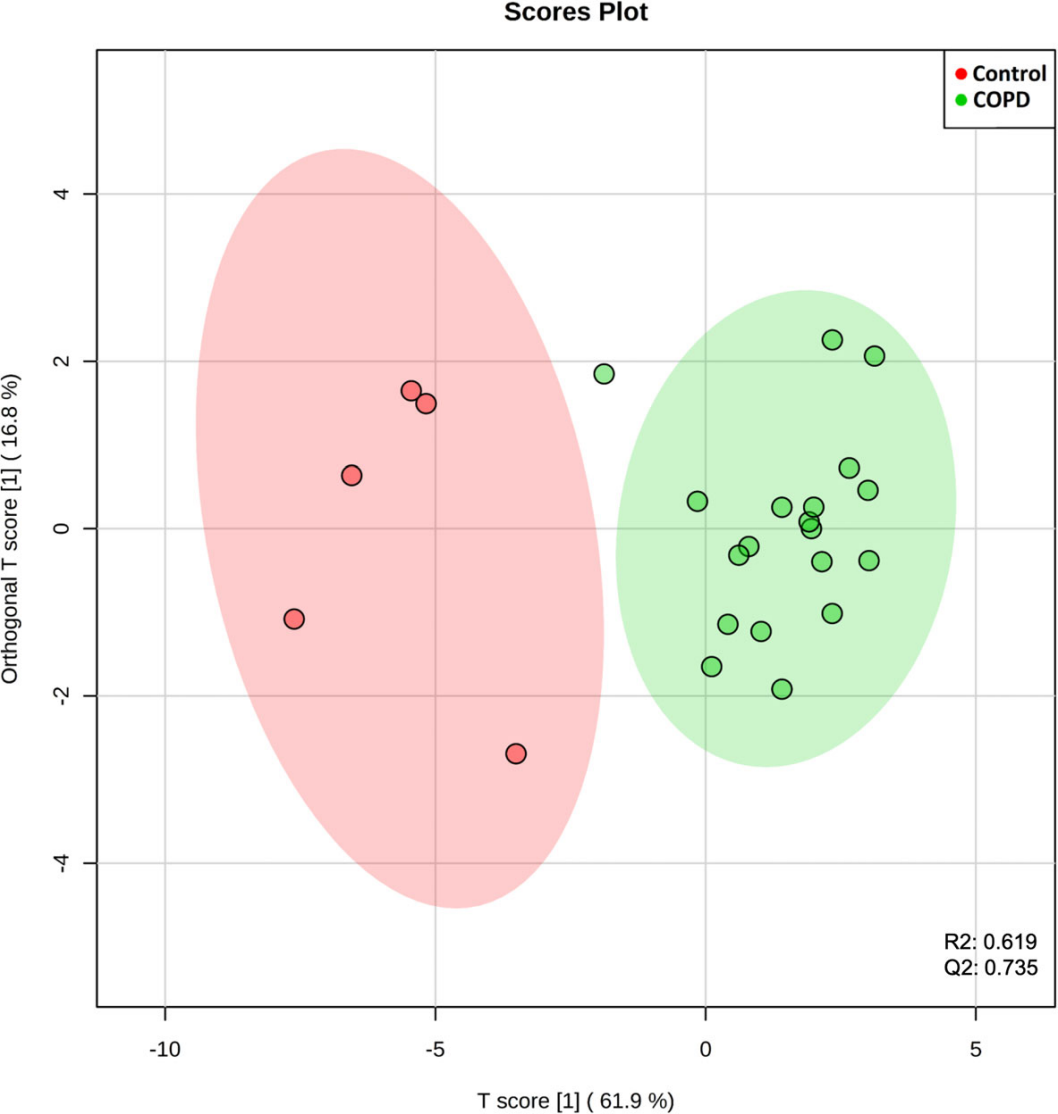
OPLS-DA evaluation of potential biomarker candidates. OPLS-DA analysis of1 0 lipid ratios candidates: R2 = 0.619 and Q2 = 0.735. Ellipses display 95% confidence regions. Serum samples from control are in red and samples from patients with COPD are in green

To validate the diagnostic potential of those biomarker candidates, a supervised multivariate data analysis (OPLS-DA) was performed to discriminate the biomarker candidates between COPD patients and control [38]. The discriminant analysis was evaluated by the cumulative modeled variation in the matrix of lipid species (R2X), the cumulative modeled variation in the matrix of the COPD samples (R2Y), and the cross-validated predictive ability (Q2) [39]. In our OPLS-DA model of 50 lipid species candidates, R2X, R2Y, and Q2 of the model were 0.111, 0.888, and 0.67 respectively; In the OPLS-DA model of 10 lipid species candidates, R2X, R2Y, and Q2 of the model were 0.242, 0.777, and 0.719; In the OPLS-DA model of 10 lipid ratio candidates, R2X, R2Y, and Q2 of the model were 0.619, 0.766, and 0.735. Q2 is used to calculate how well a variable can be predicted and estimated by cross-validation, and R2X indicates how well the variation of a variable is represented. Basically, each model with R2X > 0.5 and Q2 > 0.4 is considered as a robust model in metabolomics analysis [39]. According to these validated predictive ability (Q2) values, the lipid ratios has excellent predictive ability. As shown in Fig. 2, OPLS-DA scores plot of 10 lipid ratio candidates clearly separate the control and COPD patients. Although the model of single lipid species were not satisfied, ROC analyses were applied to further select valuable biomarkers.

**Fig. 3:**
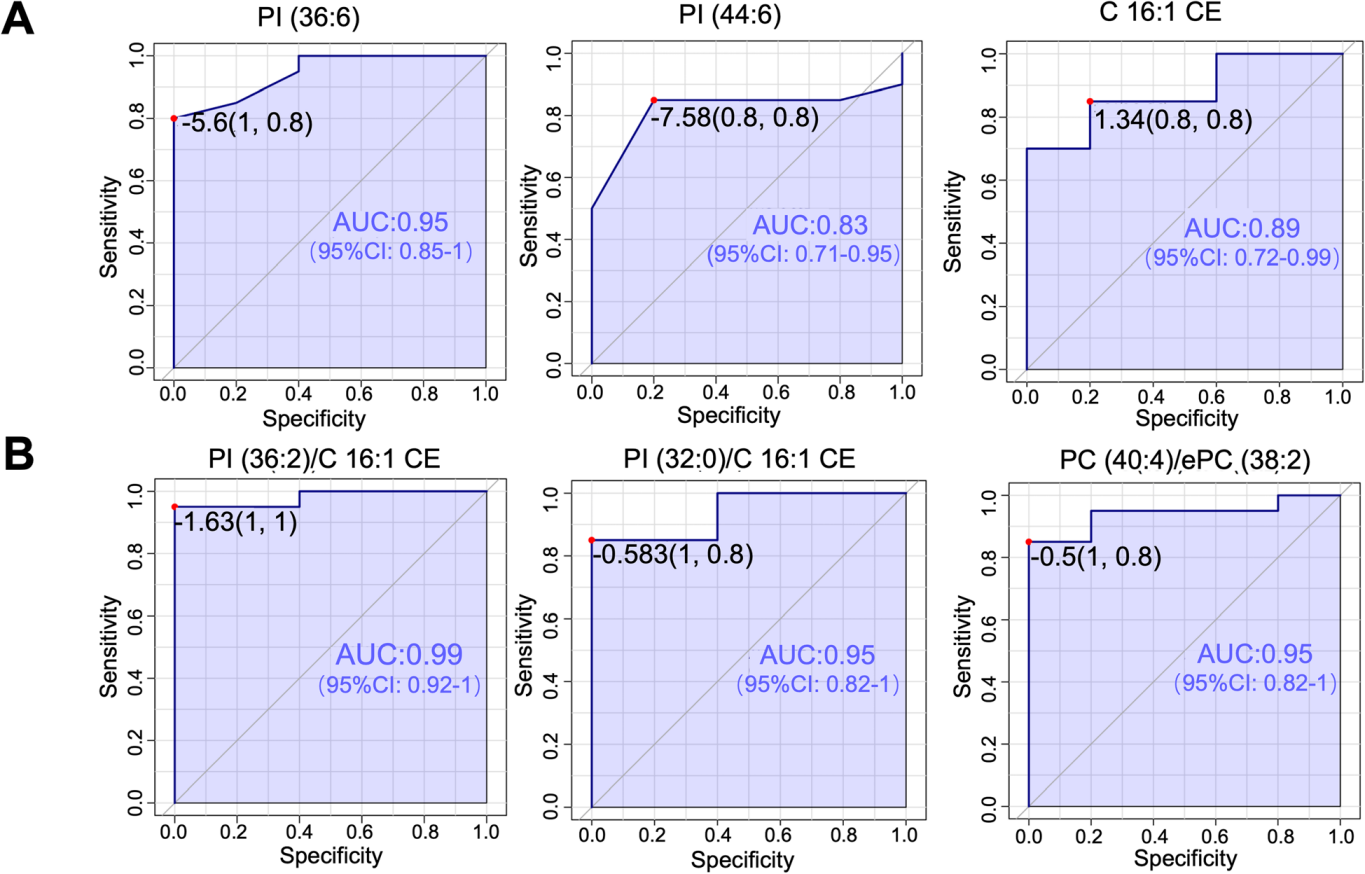
The ROC analysis from representative lipid species and lipid ratio biomarker candidates. The performance of each biomarker model was evaluated by the area under the ROC curve (AUC) and the determination of specificity (X-axis) and sensitivity (Y-axis) at the optimal cut-off point defined by the minimum distance to the top-left corner. **a**. Representative lipid species with AUC value of PC (32:1), C 16:1 CE, PI (36:6) were 0.81, 0.885, 0.95 (Top); **b**. Representative lipid ratio with AUC value of PI (36:2)/C 16:1 CE, PC (32:0)/C 16:1 CE, PC (40:4)/ePC (38:2) were 0.99, 0.95, 0.95 (Bottom)

Next, we confirmed the feasibility of using lipid ratio as COPD diagnostic markers with receiver operating characteristic curve (ROC) plot analysis. The area under the ROC curve was also utilized to assess the efficacy of the selected biomarker candidates. The results of the area under the curve (AUC), the sensitivity (true-positive rate) and specificity (false-positive rate) was calculated by MetaboAnalystR in R [28]. According to ROC analysis from 51 lipid species candidates, the 10 lipid species candidates occupied most of the top 10 highest AUC values as shown in Table 3. The possible values of AUC range from 0.5 (no diagnostic ability) to 1.0 (perfect diagnostic ability) [40]. Among these 10 lipid species, all of them had AUC values larger than 0.7, including 5 lipid species with AUC values larger than 0.8. Among the 10 lipid species, PI (36:6) had the highest AUC value 0.95, and it has significantly lower concentration (> 8 fold lower) in COPD groups. In addition, the 10 lipid ratio candidates were also evaluated by ROC analysis. As shown in Table 2, all of the lipid ratio candidates had AUC values higher than 0.9. As shown in Fig. 3, the ratio PI (38:4)/C16:1 CE had the highest AUC value at 1. Comparing with the ratio PI (38:4)/C16:1 CE in the control group, the value of PI (38:4)/C16:1 CE in patients with COPD is significant decreased with only one third. Thus, we can get high sensitivity and specificity from 4 lipid species and all 10 lipid ratios by OPLS-DA and ROC analysis, indicating the potential as serum COPD biomarkers.

**Table 3:** Top 10 lipid species biomarker candidates

Rank	Lipid Species	Mass	Element	Trend	Lipid Class	AUC	Log2FC	P value
1	PI(36:6)	872.5	C45H75O13P	↓	glycerophospholipids	0.95	−3.08	0.0057
2	C16:1 CE	640.6	C43H78NO2	↑	sterol lipids	0.88	1.54	0.0077
3	TAG(54:6) 22:6/16:0/16:0	896.8	C57H102O6N	↑	glycerolipids	0.86	1.77	0.0098
4	PI(44:6)	984.6	C53H91O13P	↓	glycerophospholipids	0.82	−1	0.0368
5	PC(32:1)	732.5	C40H78O8PN	↑	glycerophospholipids	0.8	1.42	0.0212
6	ePE(34:2)	702.5	C39H76O7PN	↓	glycerophospholipids	0.8	−1	0.041
7	TAG(54:5) 18:2/18:1/18:2	898.8	C57H104O6N	↓	glycerolipids	0.8	−1.2	0.0456
8	SM(22:0)	787.7	C45H91N2O6P	↑	gphingolipids	0.78	1.2	0.0312
9	LPE(20:2)	506.3	C25H48O7PN	↓	glycerophospholipids	0.75	−1.07	0.0432
10	ePS(38:3)	800.6	C44H82O9PN	↓	glycerophospholipids	0.73	−1	0.0445

**Fig. 4:**
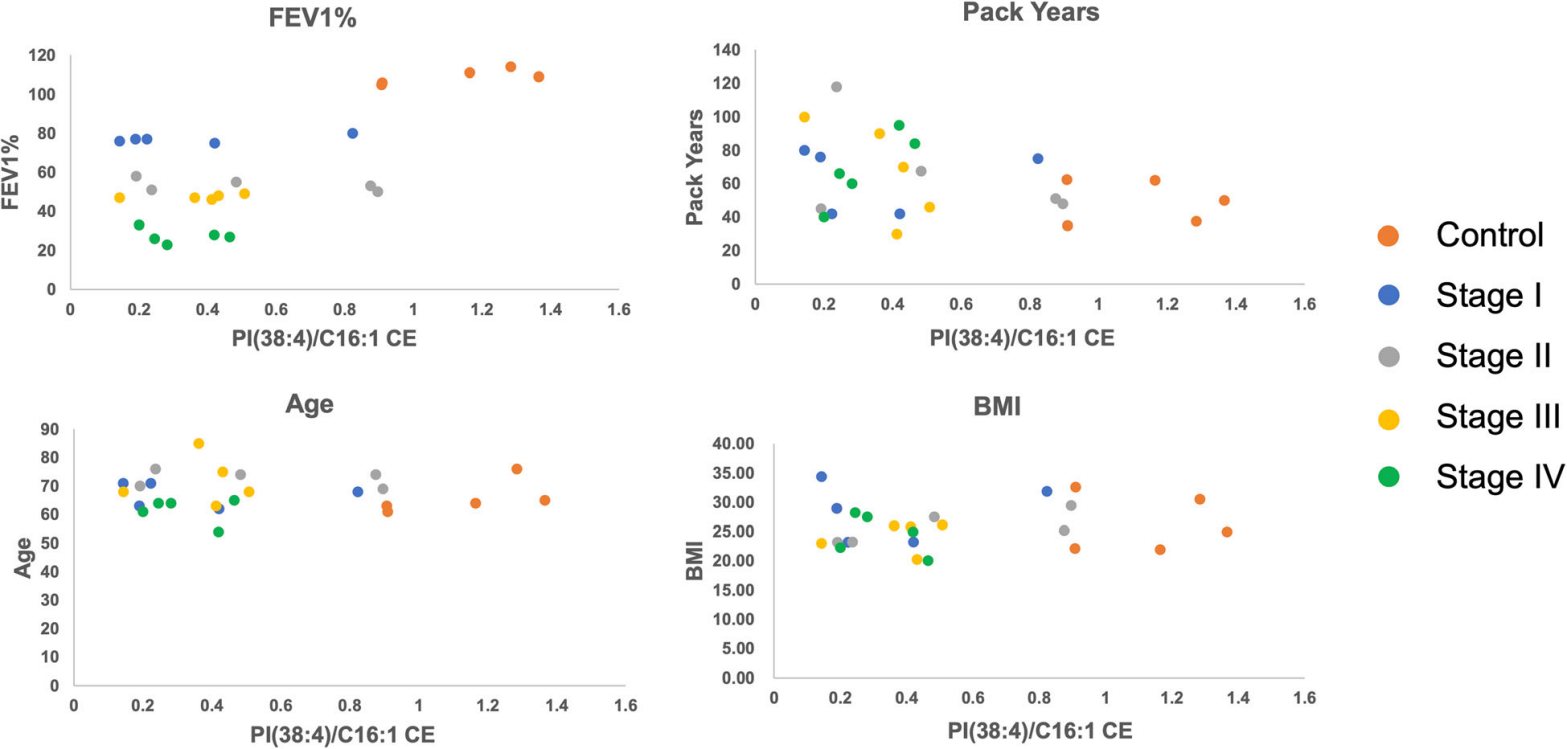
Representative lipid ratio PI (38:4)/C16:1 CE with FEV1%, Pack-Years, and Age. **a**. The lipid ratio could distinguish COPD patients and healthy people; The lipid ratio of PI (38:4)/C16:1 CE was not related to either smoking status (**b**) or the age of the patients (**c**)

Finally, we explored the ability of lipid ratio biomarkers to determine different stages of COPD. The lipid ratio PI (38:4)/C16:1 CE is shown in the spots plot in Fig. 4. As shown in Fig. 4a, the lipid ratio PI (38:4)/C16:1 CE could distinguish the control group from the patients with COPD, but it cannot distinguish different stages of COPD. Since age and smoking status are major risk factors for COPD, we were asking if the lipid ratio PI (38:4)/C16:1 CE is associated with these characteristics. Interestingly, the ratio of PI (38:4)/C16:1 CE decreases with the burden of cigarette smoking status in patients with COPD (Fig. 4b). Additionally, this lipid ratio was not affected by age (Fig. 4c). Collectively, our lipidomic results suggested individual lipid and lipid ratio signatures could be used as potential diagnostic biomarkers in future study.

## Discussion

Due to the prevalence of COPD, there is a demanding for reliable non-invasive biomarkers in diagnosing and treating patients. Blood biomarkers are relatively non-invasive and easy to obtain for this purpose [41]. Among all the blood biomarkers, blood eosinophils are the best characterized and the most applied in clinical practice [42–44]. Recent data indicates that the correlation between the COPD and the blood eosinophils is low (R^2^ = 0.35), and this correlation is significantly affected by age and the presence of hypertension [45]. Other study shows that bacteria load could lower the eosinophil counts in patients with COPD [46]. In addition, a total number of 34 blood biomarkers were assessed in the ECLIPSE cohort [47]. Among these 34 biomarkers, only fibrinogen, CC-16, and surfactant protein D were classified as stable biomarkers of baseline disease activity with low correlation coefficient [47]. Therefore, more innovative and reliable biomarkers are needed to differentiate the status of disease. Approximately 30% of COPD patients are considered obese, obesity reciprocally correlates with morbidity in patients with moderate to severe COPD [11, 48]. In order to understand the role of lipids in the development of COPD, we performed lipidomic analysis on serum from different severity levels of COPD. A total of 377 lipid compounds were identified in human serum from 25 patients. After the overall lipid profiling, using statistics and fold change filtration, a total of 10 lipid species and 10 lipid ratios were found to be significantly different between patients with COPD and controls. Together, our data suggest a lipid metabolism disorder play a unique role in the pathogenesis of COPD, and these unique lipidomic signatures need further validation for future study.

In single lipids level, 10 lipid species were selected as biomarker candidates, including six species of phospholipids, two species of glycerolipids, one species of sphingolipids, and one species of sterol lipid. Among them, three phospholipids (PC(32:1), PI(36:6), PI(44:6)), one glycerolipid TAG(54:6) 22:6/16:0/16:0, one sphingolipid (SM(22:0)), and one sterol lipid (C16:1 CE) were higher in patients with COPD comparing with the control. On the other hand, one glycerolipid TAG(54:5) 18:2/18:1/18:2, and four other phospholipids (ePE(34:2), ePS(38:3), LPE(20:2), and PS(34:0)) were lower in patients with COPD.

Lipid ratios have been proven to be a useful biomarker for several diseases, including cardiovascular disease, Alzheimer’s disease, and diabetes [49–51]. In addition, using lipid ratios as a disease indicator would carry more information and be more biologically meaningful. Our results showed that all of the ratio glycerophospholipids/sterol lipids were lower in COPD patients than in healthy controls. The ratio of two sterol lipids C16:1 CE/C19:0 CE and the ratio of two glycerophospholipids PC (40:4)/ePC(38:2) were higher in COPD groups compared with the control group. Further studies are needed to establish the biological mechanisms of these ratios in COPD patients.

In the ROC analysis, the AUC of all biomarker candidates was calculated. Generally, an AUC of 0.5 suggests no diagnostic ability to distinguish patients with and without the disease, 0.7 to 0.8 is an acceptable level, 0.8 to 0.9 is an excellent level, and more than 0.9 is an outstanding level [52]. Based on this criteria, for single lipid biomarker candidates, lipids including PS(34:0), ePS(38:3), LPE(20:2), ePE(38:6), SM(22:0) are acceptable biomarker candidates; ePE(34:2), PC(32:1), PI(44:6), C16:1 CE are excellent biomarker candidates; and PI(36:6) is outstanding biomarker candidate. For lipid ratio biomarker candidates, all of them are outstanding biomarker candidates. The AUC values of lipid ratio biomarker candidates showed higher sensitivity (true-positive rate) and specificity (false-positive rate) for distinguishing COPD objects from controls (Fig. 4). Here we applied AUC value above outstanding level (AUC > 0.9) as cut off point, all of the 10 lipid ratios and 4 lipid specie (AUC > 0.9) are qualified as potential biomarkers in diagnosing COPD disease in the future. However, the biological functions of these biomarker candidates need to be validated before the confirmation of useful COPD biomarkers.

Glycerolphospholipids, which are the main lipid class identified in this study, are highly enriched within the pulmonary airway surfactant. A surfactant disorder was reported to be related to the pathogenesis of COPD [53]. PI consisted of an inositol group and two fatty acid chains linked by a glycerol backbone. In a previous report, the upregulation of phosphatidylinositol-3 kinase (PI3K) generated extra oxidative stress on COPD patients [54]. The lower production of PI in our study could partially due to the activation of PI3K pathway in patients with COPD. Pulmonary surfactant is a complex mixture consisting of phosphatidylcholine (PC), neutral lipids, and specific proteins [8, 9]. It is essential for normal lung function because it reduces surface tension at the air–liquid interface of lung alveoli. Surfactants deficiency might be related to the development of COPD [10, 11]. PC16:0/16:0 was reported to be a key composition of pulmonary surfactant [55]. PC 16:0/18:0 was also reported to represent around 1% of surfactant phospholipid [56]. Besides, the higher level of phosphatidylcholine hydroperoxide was found to be associated with the increase of pulmonary artery pressures, which is important in the pathogenesis of COPD [57]. Previous study indicated PC is its main component of surfactant, while proteins and other phospholipids contribute to the dynamic properties and homeostasis of alveolar surfactant [13]. The PC profiling from the patients with different stages of COPD were demonstrated in Supplemental Figure 2. We found PC (16:0/18:1) percentage is positively correlated with the severity of COPD. While PC (16:0/18:2) and PC (16:0/20:2) is negatively correlated with the severity of COPD. This data suggests that alteration of PC in plasma are pronounced in the pathogenesis COPD. As pulmonary surfactant deficiency was closely associated with COPD [14], the change of PC 32:1 in COPD patients in our results could be another indicator of surfactant composition change. Due to small sample size, our data did not reach the significant difference. Therefore, PC labeling signature need to be validated in large dataset using stable isotope standards for potential biomarker in the future.

However, PE was reported to increase in COPD patients’ serum or sputum by previous lipidomics study [15, 58], which was not identified in our study. Phosphatidylserine (PS) comprises a minor percentage of a phospholipid, but it has an important effect on cell signaling and blood coagulation [59]. Interestingly, PS and ePS were higher expressed in our results, which were not observed in previous studies on COPD patients in our knowledge. Although COPD was not reported to have a direct influence on Triacylglycerolipid (TAG), we observed a unique labeling pattern of TAG in patients with COPD. Among them, TAG(54:5) 18:2/18:1/18:2 was lower while TAG(54:6) 22:6/16:0/16:0 was higher in patients with COPD, suggesting these labeling patterns may play essential roles in the development of COPD.

Sphingolipids play an essential role in the development of COPD [10, 60]. Smokers and COPD patients have elevated levels of ceramides in their lungs. Accumulating data indicate that tobacco smoke induces pulmonary and vascular cell apoptosis via ceramide production by alveolar macrophages [10, 61–63]. Sphingomyelins (SM), including phosphoethanolamines and ceramides, are the main class of sphingolipids. Neutral sphingomyelinase-2 (nSMase-2), which hydrolyzes sphingomyelin to produce ceramide, is elevated in small airways cells and alveolar macrophages from COPD patients compared with the control [64]. Consistent with these data, we found a higher level of sphingomyelin (SM, 22:0) in COPD patients. Thus, identification of specific lipid regulations may yield new insight for diagnosis and treatment.

**Fig. 5:**
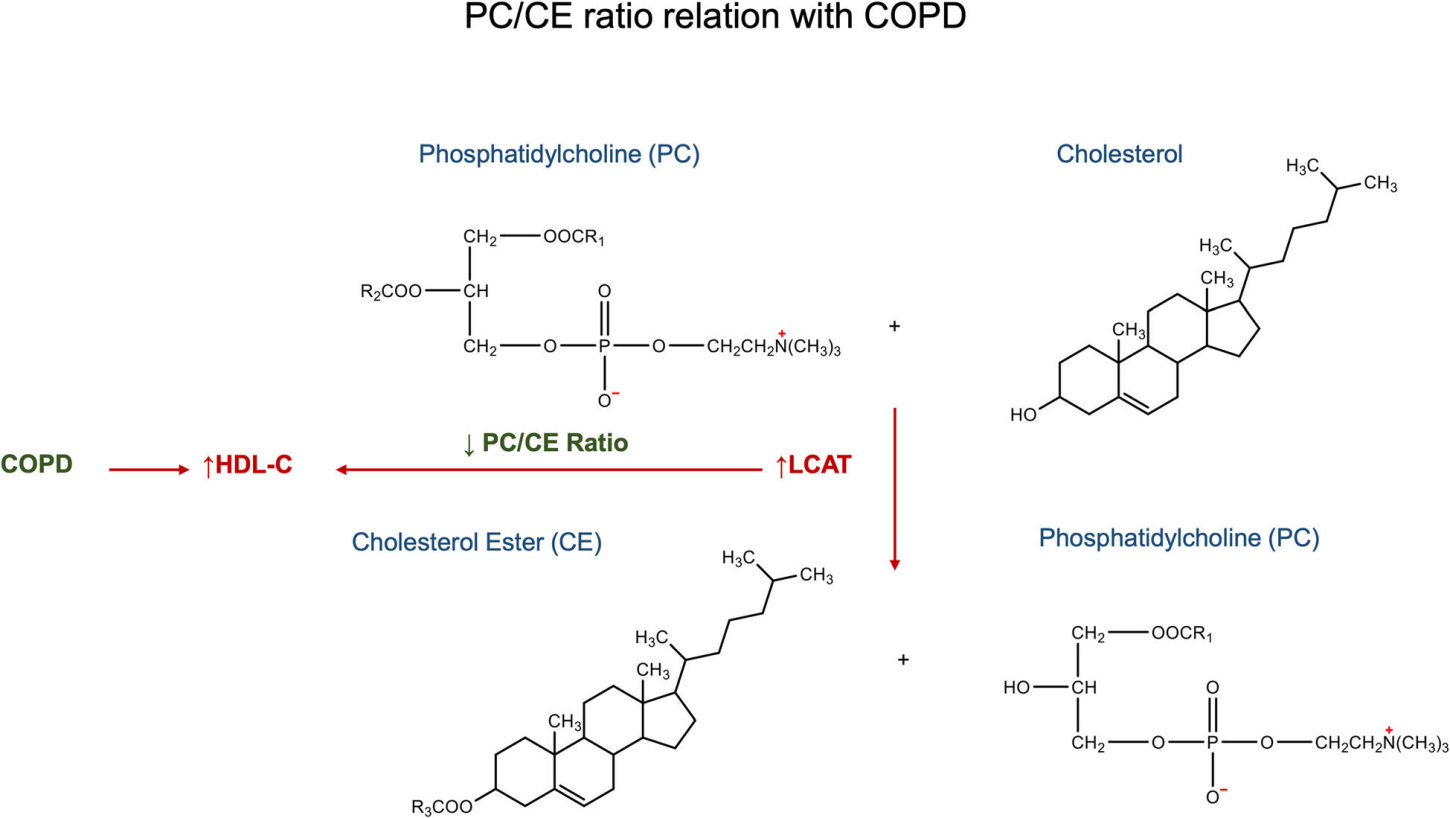
Potential mechanistic scheme of PC/CE ratio identified in sera of patients with COPD

The high hydrophobicity of cholesterol ester (CE) leads the esterified cholesterol moving from the surface into the center within discoidal high-density lipoprotein (HDL), which will transform discoidal HDL into spherical HDL [65]. This transformation leads HDL to higher cholesterol carrying capacity in the blood. Consistent with previous data [66], the major format of cholesterol ester is C18:2 CE as shown in Supplemental Table 1. Patients with very severe COPD have a much higher level of cholesterol [33, 35]. Interestingly, our results indicated the ratio of C16:1 CE in patients with COPD is about 3 times higher than that in healthy controls. Biosynthesis pathways of lipids usually involve two or more lipid species and related enzymes. Therefore, changes in these biosynthesis pathways can be reflected by a specific lipid ratio. Lecithin cholesterol acyltransferase (LCAT) is a key enzyme for the production of cholesteryl esters in plasma, which exerts on the formation and maturation of HDL-C [67, 68]. Ratios of glycerophospholipids/sterol lipids in our study consist of two major classes, PI/CE and PC/CE. PC/CE ratio is highly associated with the function of lecithin cholesterol acyltransferase (LCAT), which can hydrolyze phosphatidylcholine and transfer the free cholesterol to cholesterol ester (CE). After esterification of cholesterol, the higher hydrophobicity of CE would enhance the capacity of lipoproteins. The correlation between the concentration of HDL and the status of COPD is not very conclusive. Clinical studies on men with more than 10 years of smoking history showed that smokers had lower plasma HDL fractions 2 and 3 [37]. Another meta-analysis indicated that smokers had significantly higher concentrations of cholesterol and LDL, and the lower concentrations of HDL in the serum [36]. On the contrary, other studies showed patients with COPD tend to have higher levels of high-density lipoprotein cholesterol (HDL-C) [69]. As previously described in the discussion, COPD is associated with an increasing level of HDL-C, which refers to the elevation of LCAT. Therefore, the decreasing PC/CE ratio in COPD patients is possibly due to the increased function of LCAT, resulting in the elevation of HDL-C (Fig. 5). In addition, PI/CE ratio occupied the top 2 in the AUC value rank of 10 lipid ratio biomarker candidates. Although the PI/CE ratio does not involve pathways like the PC/CE, PI has been shown to enhance the mobilization of cellular sterol and promote a rapid clearance of both cholesterol and cholesterol ester from the plasma in vivo [70]. Phosphoinositide-3-kinase-delta (PI3K-δ) is activated in COPD and targeting PI3K-δ mimic the effects of could reverses corticosteroid resistance in animal model of COPD [71].

Finally, correlation analysis was performed to explore the possible effects of age, BMI, stages, FEV1% and smoking pack years, on the identified biomarkers. All the correlation analysis were shown as scatter plot in supporting information (Supplemental Figure 3–12). Through the correlation analysis, all the lipid ratios could only discriminate the healthy people from the COPD patients, but they were not affected by age, BMI, stages, FEV1%. Interestingly, the lipid ratios showed differences with the smoking exposure in patients with COPD, while the healthy people had relative low pack years among all samples. Since smoking is a main cause of COPD, it is reasonable for these potential biomarkers being affected by smoking pack years. Although the results are promising, limitations still exist in our study. Due to the limited sample size and the sample size difference between control and disease groups, further efforts need to validate in a large independent dataset. Besides, since the instrument limitation, targeted lipid study cannot be performed, which could further validate the results.

## Conclusions

In conclusion, our study has identified potential biomarkers for the diagnosis of COPD and acquired a vital clue of lipid metabolism in COPD patients. Our results discovered 4 individual lipid molecules and 10 lipid ratios being able to differentiate COPD from controls. Among these biomarkers, PI, PC, CE and their ratios PI/CE, PC/CE are biologically significant to COPD. LCAT could be a potential target for COPD. Future efforts would be focused on validating these biomarkers using a large sample size and targeted lipidomics approach. Finally, a panel of stable biomarkers will enable us to better predict response to therapy and prognosis.

## Supplementary information

**Additional file 1: Supplemental Figure 1**. Representative of MS/MS example ion spectrum for 798.5 and 496.3 m/z using negative ESI mode.

**Additional file 2: Supplemental Figure 2**. Phosphatidylcholine molecular species distribution across the severity of COPD patients (red is up; green is down).

**Additional file 3: Supplemental Figure 3–12**. Correlations between the lipid ratios and FEV1, Smoking Pack Years, Age and BMI.

**Additional file 4.**

**Additional file 5.**

## Data Availability

The datasets used and/or analyzed during the current study are available from the corresponding author on reasonable request.

## Abbreviations

AUC–ROC: The area under the receiver operating characteristics curve
CE: Cholesterol ester
COPD: Chronic obstructive pulmonary disease
ESI-MS: Electrospray ionization mass spectrometry
ePC: Ether-linked phosphatidylcholine
ePE: Ether-linked phosphatidylethanolamine
ePS: Ether-linked phosphatidylserine
HDL: High-density lipoprotein
LPA: Lysophosphatidic acid
LPC: Lysophosphatidylcholine
LPE: Lysophosphatidylethanolamine
LPG: Lysophosphatidylglycerol
OPLS-DA: Orthogonal projections to latent structures discriminant analysis
PC: Phosphatidylcholine
PE: Phosphatidylethanolamine
PG: Phosphatidylglycerol
PI: Phosphatidylinositol
PS: Phosphatidylserine
ROC: Receiver operating characteristics
SM: Sphingolipid
TAG: Triacylglycerolipid
