# Supplementary material for: Identification of lipid biomarker from serum in patients with chronic obstructive pulmonary disease
Source: Respir Res. 2020 Sep 21;21:242. doi: 10.1186/s12931-020-01507-9 (PMC7507726; doi:10.1186/s12931-020-01507-9)

Supplemental Figure 2

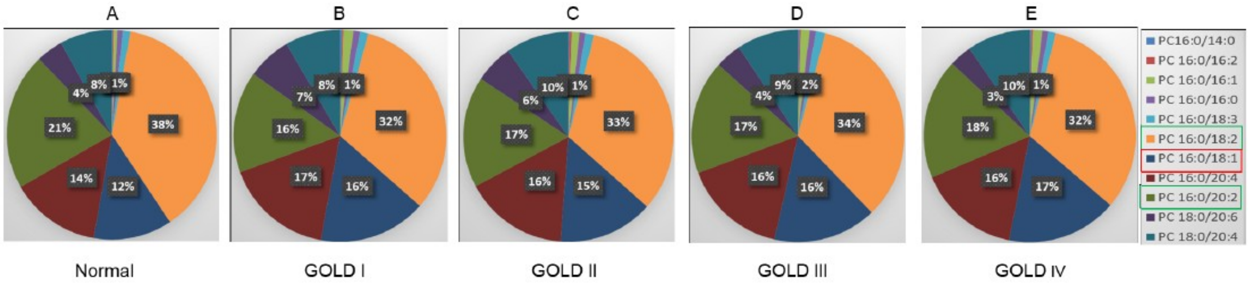

Supplement: Supplementary file 2 [file 12931_2020_1507_MOESM2_ESM.docx]
