# Supplementary material for: Identification of lipid biomarker from serum in patients with chronic obstructive pulmonary disease
Source: Respir Res. 2020 Sep 21;21:242. doi: 10.1186/s12931-020-01507-9 (PMC7507726; doi:10.1186/s12931-020-01507-9)

Supplemental Figure 3

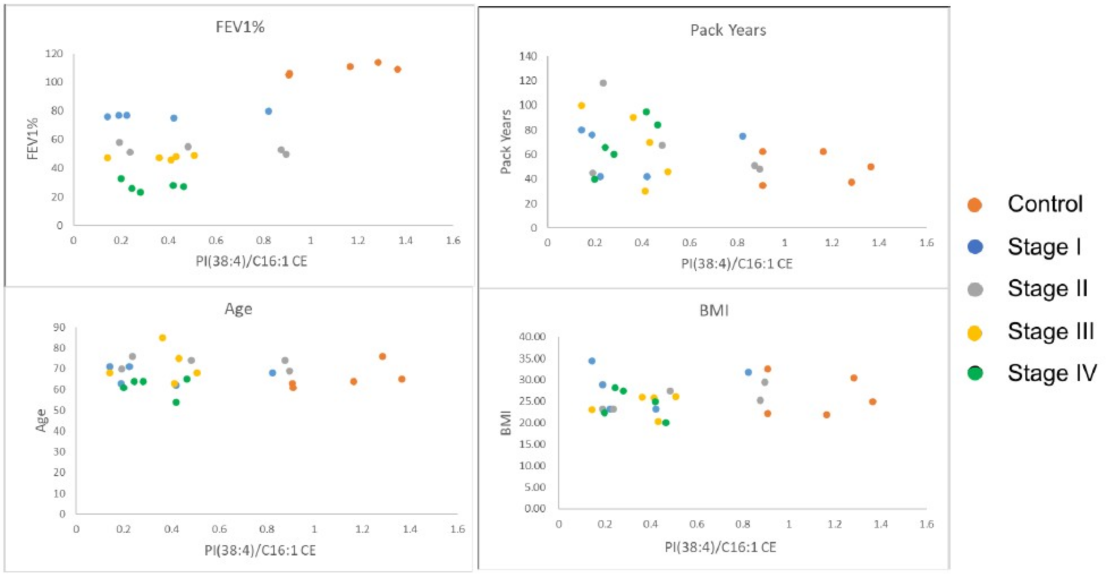

Supplemental Figure 4

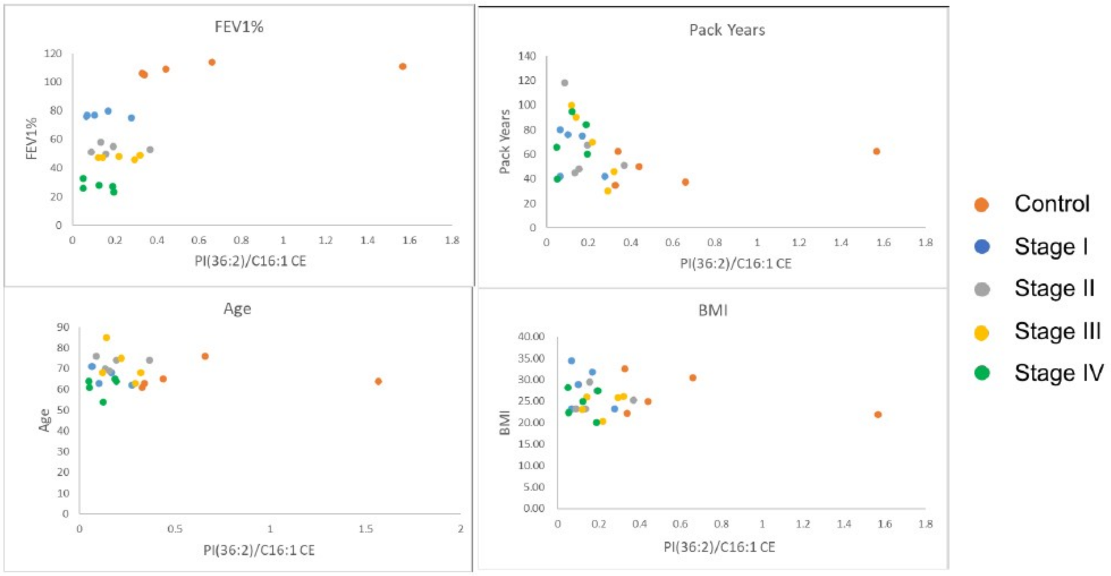

Supplemental Figure 5

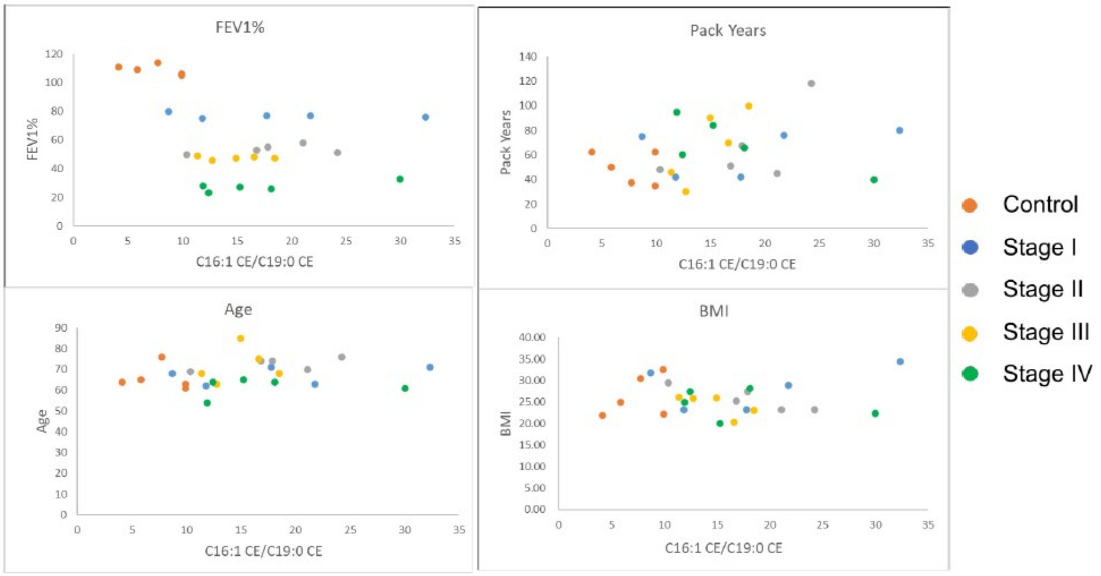

Supplemental Figure 6

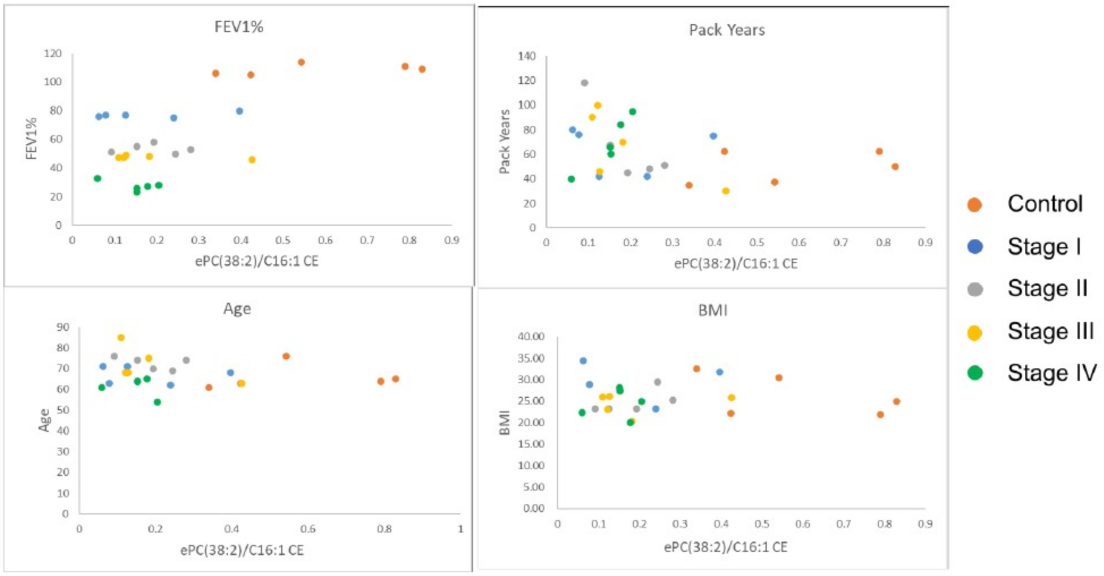

Supplemental Figure 7

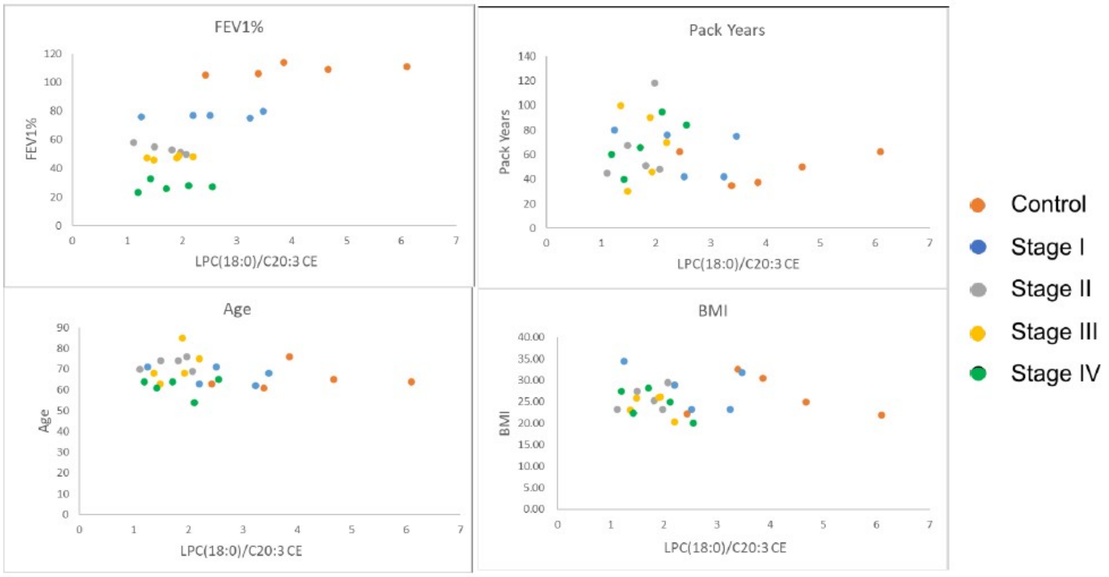

Supplemental Figure 8

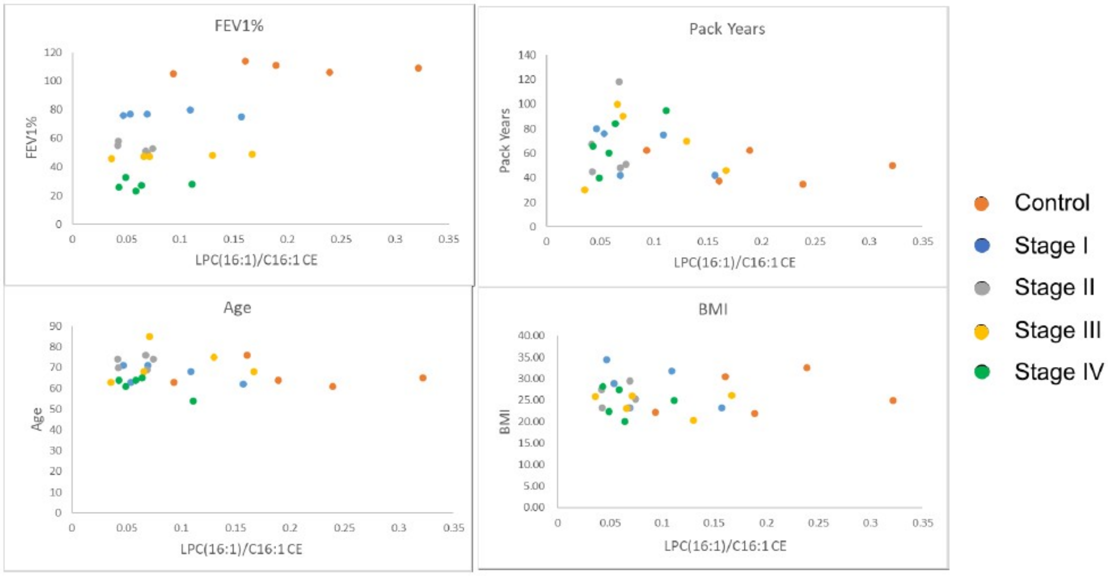

Supplemental Figure 9

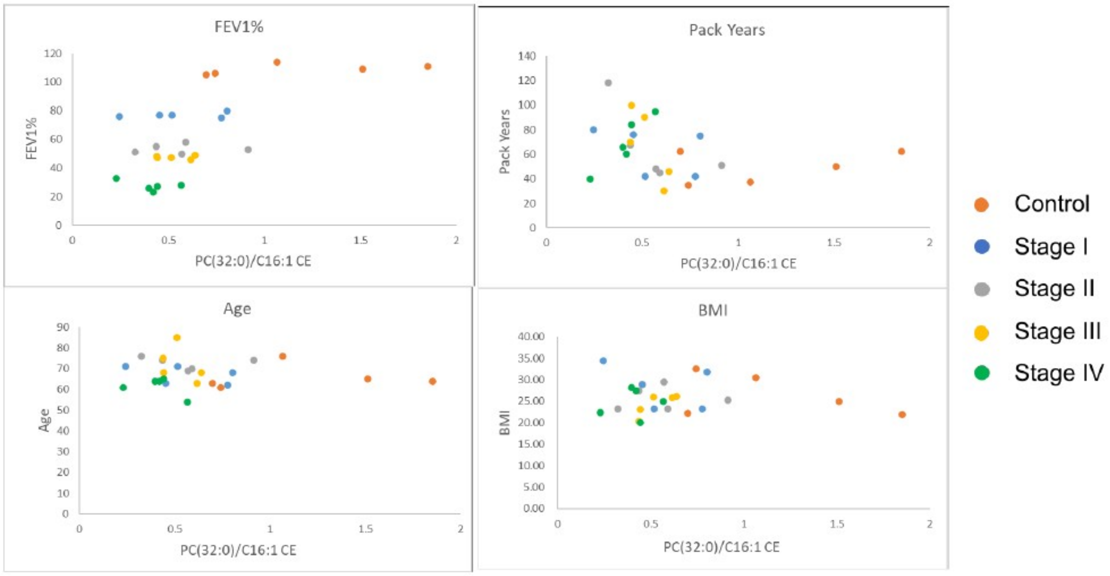

Supplemental Figure 10

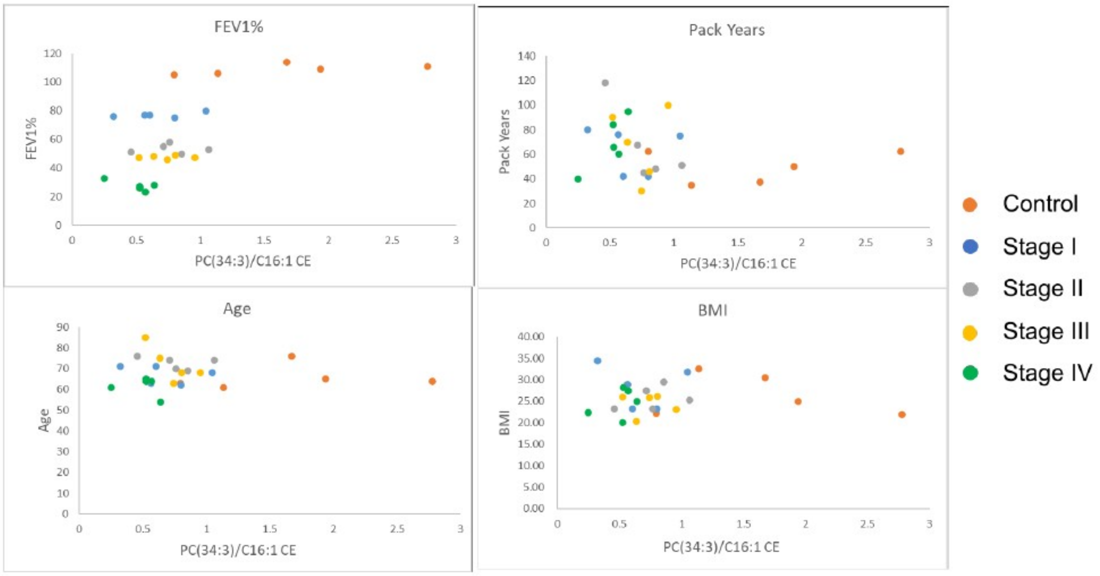

Supplemental Figure 11

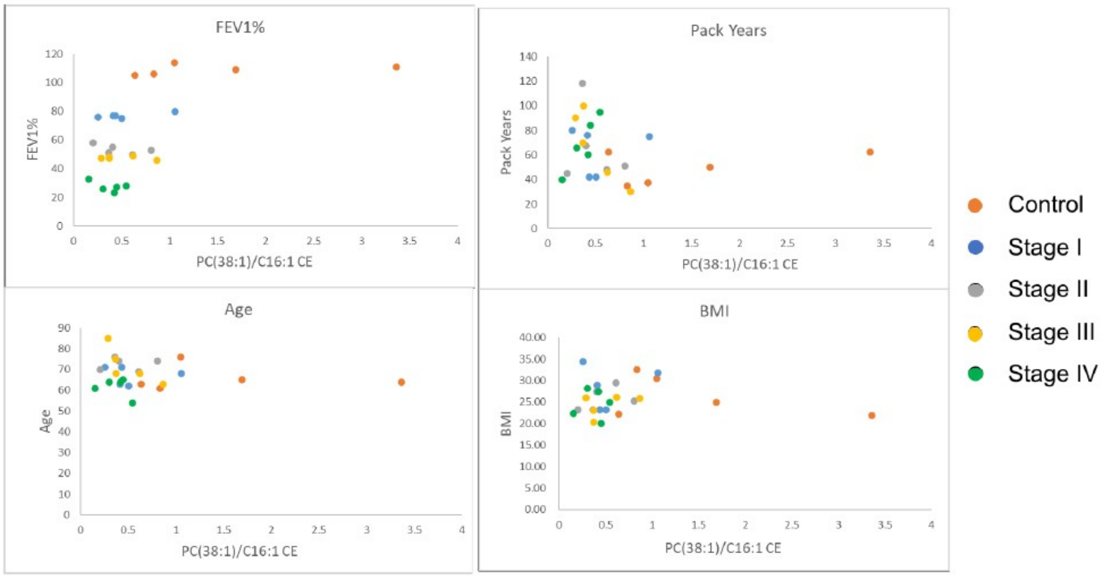

Supplemental Figure 12

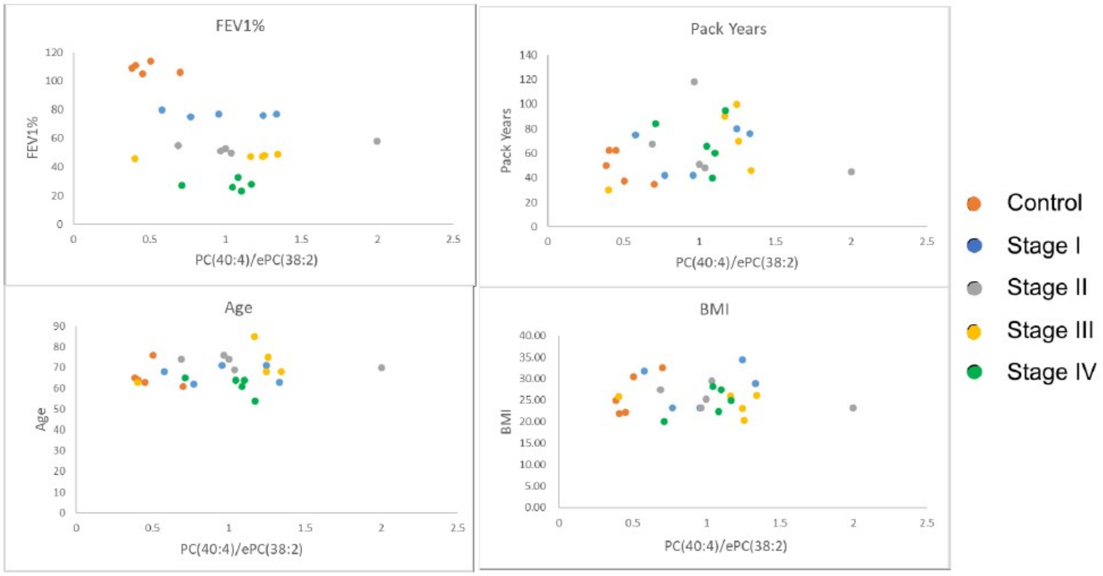

Supplement: Supplementary file 3 [file 12931_2020_1507_MOESM3_ESM.docx]
